# Sargassum incisifolium and Ulva spp metabolites activity and their molecular dynamics simulation against Fusarium oxysporum 14-alpha-demethylase

**DOI:** 10.1016/j.btre.2025.e00919

**Published:** 2025-08-20

**Authors:** Omolola Aina, Adewale O. Fadaka, Daniel Watson, Cecilia Y. Ojemaye, Denzil R. Beukes, Kudakwashe Nyambo, Kudzanai Tapfuma, Vuyo Mavumengwana, Nicole R. S Sibuyi, Marshall Keyster, Ashwil Klein

**Affiliations:** a Plant Omics Laboratory, Department of Biotechnology, University of the Western Cape, Robert Sobukwe Road, Bellville 7530, South Africa; b Department of Science and Innovation/Technology Innovation Agency Nanotechnology Platform, Department of Biotechnology, University of the Western Cape, Bellville, 7535, South Africa; c Division of Clinical Pharmacology, Department of Medicine, Faculty of Health Sciences, University of Cape Town, Cape Town, South Africa; d Environmental Humanities South, University of Cape Town, South Africa; e School of Pharmacy, University of the Western Cape, Bellville 7535, South Africa; f DST-NRF Centre of Excellence for Biomedical Tuberculosis Research; South African Medical Research Council Centre for Tuberculosis Research; Division of Molecular Biology and Human Genetics, Faculty of Medicine and Health Sciences, Stellenbosch University, Tygerberg 7505, Cape Town, South Africa; g Environmental Biotechnology Laboratory, Department of Biotechnology, University of the Western Cape, Robert Sobukwe Road, Bellville 7530, South Africa

**Keywords:** Antifungal, 14- α -demethylase, *F. oxysporum*, *Sargassum incisifolium*, seaweed, molecular dynamics simulation, *Uva spp*

## Abstract

Fusarium oxysporum, a major agricultural pathogen, poses severe risks to crops worldwide. With increasing resistance to conventional antifungal agents, there is an urgent need for alternative treatments. Seaweeds such as *Ulva spp.* and Sargassum incisifolium are promising sources of bioactive compounds that may offer novel antifungal properties. This study investigates the antifungal activity of acetone extracts from *Ulva spp.* and Sargassum incisifolium against *F. oxysporum*, with the goal of identifying specific bioactive compounds responsible for this activity and evaluating their effectiveness quantitatively. We prepared acetone extracts from both seaweed species and assessed their antifungal activity using a series of in vitro assays. The total phenolic content (TPC) and antioxidant capacity were determined for each extract. LC-qTOF-MS/MS was employed for phytochemical profiling, while molecular docking and molecular dynamics simulations were used to predict interactions between identified compounds and the 14-alpha-demethylase enzyme of *F. oxysporum*. The TPC was 2.72±0.009 and 2.23±0.009 GAE/mg dry weight for *Ulva spp* and *S. incisifolium*. Additionally, significant antioxidant activity was observed, with IC50 values of 8.38±0.06 µg/mL for *Ulva spp* and 8.01±0.07 µg/mL for *S. incisifolium*, which are comparable to ascorbic acid (5.23±0.04 µg/mL). Phytochemical analysis revealed high levels of terpenoids, phenolics, and fatty acids. In molecular docking, compounds such as medicocarpin, corynanthine, and merulinic acid demonstrated strong binding affinities (binding energies ≤ -7.5 kcal/mol). Molecular dynamics simulations confirmed stable interactions over 100 ns, with medicocarpin exhibiting the most stable binding profile. The study demonstrates that acetone extracts of *Ulva spp.* and *S. incisifolium* possess significant antifungal activity against *F. oxysporum*. Medicocarpin, in particular, emerged as a promising candidate for further development as an antifungal agent. These findings underscore the potential of seaweed-derived compounds as antifungal agents against fungal pathogens and highlight the need for further investigation into their practical applications in plant disease management. Specifically, Medicocarpin emerged as a promising *in silico* candidate, warranting further experimental validation.

## Highlights

• Untargeted LC-qTOF-MS/MS analysis identified diverse classes of bioactive metabolites, including terpenoids, alkaloids, fatty acids, and phenolics from seaweed plants.
• Medicocarpin, corynanthine, and merulinic acid showed inhibitory potentials with strong binding affinities against *F. oxysporum* 14-α-demethylase.
• Medicocarpin, corynanthine, and merulinic acid also indicated stable interactions and structural integrity of the protein-ligand complexes suggesting their anti-fungi activity.
• The study supports the potential of seaweed-derived phytochemicals as novel antifungal agents targeting *F. oxysporum* 14-α-demethylase.
• Medicocarpin, corynanthine, and merulinic acid demonstrated significant promise for further investigation as antifungal therapeutics.
• These highlights provide a comprehensive overview of the key findings and contributions of the research, emphasizing the potential of seaweed-derived compounds in antifungal applications.

## 1 Introduction

Plant diseases significantly threaten food security, drastically reducing food availability for human consumption [1]. They are estimated to reduce the yield of essential staple crops by 30 %, coupled with economic losses worth hundreds of billions of dollars annually [2]. Among various disease-causing pathogens in plants, fungi are the most prevalent, accounting for 70-80 % of these diseases [3]. Additionally, historical famines, such as the Irish potato famine between 1846-1850 and the 2009 wheat blast outbreak in Brazil, were both caused by fungi [4,5]. An estimated 125 million tons of the top five food crops, namely wheat, rice, potatoes, soybeans, and maize, are lost to fungal diseases, potentially depriving 600 to 4,000 million people worldwide of their daily caloric intake for a year [6]. These losses impede the advancement of sustainable food production [3]. Unlike prior studies focusing solely on crude seaweed extracts, this study integrates LC-qTOF-MS/MS metabolite profiling with target-specific in silico modeling against the 14-α-demethylase enzyme of F. oxysporum.

Fusarium is one of the most destructive pathogenic fungi, possessing the ability to infect nearly all economically important crop plants and cause diseases such as dermatitis, onychomycosis, and keratitis in humans [7,8]. Additionally, it is notorious for its mycotoxin production, which poses serious health risks to humans and animals [9]. The effectiveness of *Fusarium* spp. as a phytopathogen is due to several factors, including its broad host range, extensive genetic variability, diverse dispersal methods, absence of external symptoms on the host plant at the early infection stage, and its ability to adapt to a wide range of environments [10].

Numerous strategies have been used to mitigate Fusarium infestations, including genetically modified resistant cultivars, cultural practices to limit conidia proliferation, and applying chemical fungicides. Nevertheless, these strategies have their respective limitations [8,11]. For instance, the development of resistant cultivars is both financially taxing and time-intensive, with instances of fungi eventually overcoming host plant defenses [12,13]. Chemical fungicides remain the most effective means of control. Still, they raise significant concerns related to ecosystem contamination, adverse effects on non-target organisms such as soil microbiota, humans, and animals, and the necessity for frequent reapplication, contributing to an environmental chemical buildup [14]. Consequently, as awareness of cost-effective and sustainable agricultural practices continues to grow, there is a growing interest in adopting biological control methods, which encompass the utilization of microorganisms and natural bio-actives from plants and marine organisms [13,15].

Seaweed or macroalgae are photosynthetic plant-like organisms found in the marine environment. They constitute an essential part of the ecosystem, where they perform numerous roles, including being a source of food and oxygen, reducing the ocean's acidity, and providing shelter for other aquatic organisms [16]. In their harsh marine environments, seaweed is exposed to various stressors such as extreme temperatures, high salinity, intense UV radiation, and predation. To survive these extremities, they have developed remarkable coping mechanisms, one of which involves the synthesis of bioactive molecules such as phenolic compounds, osmolytes, and terpenoids [17]. These molecules play a crucial role in the seaweed's ability to thrive in challenging environments [18,19].

For example, a study by Le Lann and colleagues reported that *Sargassum* species accumulate phenolic compounds as a chemical defence mechanism against harsh environmental conditions such as herbivory, epiphytic colonization, and ultraviolet radiation that are particularly intense in wave-exposed coastal environments [20,21]. In addition to phenolics, seaweeds are known to biosynthesize halogenated secondary metabolites, such as furanones and halogenated anones, which exhibit potent antimicrobial and antifouling activities [22,23]. These compounds play a critical role in inhibiting the growth of biofilm-forming bacteria and the attachment of epiphytes, thereby protecting seaweed surfaces from microbial infection and fouling, which are common challenges marine organisms face [24]. These chemically mediated defence strategies ensure seaweeds' survival in their natural habitats and highlight their potential as a valuable source of bioactive compounds [25].

Furthermore, previous studies have documented the antifungal properties of extracts derived from different seaweed taxa, including red, green, and brown, against various phytopathogenic fungi [[26], [27], [28]]. For example, red seaweed *Gracilariopsis persica* effectively inhibited the growth of *Aspergillus niger, Botrytis cinerea, Pyricularia oryzae*, and *Penicillium expansumin* in a dose-dependent manner, with 1000 µg/mL causing complete inhibition of the fungal growth. The antifungal activity was attributed to bioactive compounds, such as phenolic compounds identified via gas chromatography mass spectrometry in the crude extract [29]. Additionally, Valverde and Tyśkiewicz demonstrated the antifungal properties of some brown seaweeds, *Ascophyllum nodosum, Eclonia maxima and,* against *Penicillium digitatum, Botrytis cinerea, F. culmorum and F. oxysporum*. They reported that the fungicidal effect of these algae might be associated with the interactive effect of numerous phytochemical constituents such as polysaccharides, carotenoids, and polyphenols [30,31].

Several studies have demonstrated that extracts from *Sargassum spp*. (brown seaweed) and *Ulva spp*. (green seaweed) exhibit significant antifungal activity against *F. oxysporum*. For example, Toledo and Ambika reported that extracts of *S. muticum* and *S. myricocystum* effectively inhibited both mycelial proliferation and spore germination of *F. oxysporum*, with the antifungal activity attributed to bioactive secondary metabolites, including terpenes and phenolic compounds [32,33]. Similarly, extracts from *S. cinereum* exhibited strong antagonistic effects against *F. oxysporum*, likely due to the presence of compounds such as hexadecanoic acid, dimethylocta-1,6-dien-3-ol, 1-methoxy-4-(2-propenyl)benzene, octadecanoic acid, and its methyl ester derivative [34,35]. In the case of green seaweeds, *U. fasciata* and *U. lactuca* also showed inhibitory effects on mycelial growth of *F. oxysporum*, which were attributed to the presence of antifungal compounds including phenol, phthalic acid, and γ-sitosterol [36].

Conventional methods for identifying and investigating the bioactivity of metabolites from crude extracts entailed a series of steps, including isolation, fractionation, and *in vitro* testing of all fractions obtained from the crude extracts for bioactivity [37]. These techniques are expensive, laborious, and time-consuming. However, in modern times, innovative methods such as *in silico* modeling allow researchers to predict the bioactivity of phytochemicals within crude extracts, streamlining the process by identifying only the compounds with potential bioactivity for further isolation and *in vitro* testing [38]. These approaches offer time savings, reduced labor intensity, and lower costs [39].

*In-silico* approaches can be used to predict the interactions between potential antifungal drugs and their molecular targets, such as fungal enzymes or proteins involved in essential cellular processes [40]. There are several targets through which an antifungal compound can inhibit fungal growth. Such mechanisms have been considered significant antifungal targets for decades [41]. Examples include succinate dehydrogenase enzyme, β-tubulin protein, cytochrome-b protein, and 14-α-demethylase enzyme [42].

The 14-α-demethylase protein is an enzyme belonging to the cytochrome P450 monooxygenase superfamily. It plays a pivotal role in fungal metabolism as the major enzyme involved in the biosynthesis of ergosterol [43]. Ergosterol is a fungal-specific sterol crucial in maintaining membrane integrity, fluidity, and the proper function of membrane-bound enzymes. By inhibiting 14-α-demethylase, antifungal agents impede ergosterol synthesis, hindering fungal growth and ultimately leading to cell death [44]. Furthermore, 14-α-demethylase is one of the most preferred antifungal targets in drug discovery due to the uniqueness of the ergosterol synthesis pathway in fungi, which reduces the possibility of negative impacts on non-target organisms.

The present study aims to tentatively identify the phytochemical composition of the acetone extract of *Ulva spp* and *S. incisifolium* using untargeted LC-qTOF-MS/MS*.* Additionally, molecular docking was carried out to predict the binding scores of the identified compounds against the demethylase enzyme, and molecular dynamics simulation was performed to determine the dynamic stability of the phytocompound-demethylase protein.

## 2 Materials and methods

### 2.1 Materials

The chemicals used in the present study such as Folin-Ciocalteu reagent, acetone, and 2, 2-diphenyl-1-picrylhydrazyl (DPPH), ascorbic acid, Sodium carbonate (Na_2_CO_3_), gallic acid, methanol, formic acid, and ammonium formate were obtained from Sigma–Aldrich St Louis, Missouri, USA); papers discs were from Whatman (Masiya laboratories, South Africa). The spectrophotometer (FLUOstar® Omega, BMG LABTECH, Ortenberg, Germany) was used to measure the samples' absorbance.

### 2.2 Collection and authentication of plant materials

*S. incisifolium* was collected from Kelly's Beach, Port Alfred (-33.611650, 26.890295), while the Ulva spp was collected from False bay beach (34°13′57.9, 18°28′36.5) in Cape Town (South Africa). Samples were carefully washed in distilled water to remove marine epiphytes and other debris and stored at −20°C for downstream analysis.

### 2.3 Experimental analysis

#### 2.3.1 Preparation of plant extracts

The extraction process was performed as described previously following a protocol by Truong, Nguyen [45] with minor modifications. Briefly, 50 g of each of the seaweed samples (*Ulva spp* and *S.incisifolium*) were extracted with water: acetone mixture (1:4 ratio). The mixtures were extracted for 48 h at room temperature with shaking at 150 rpm. This process was repeated thrice to achieve exhaustive extraction of the plant materials. Next, crude extracts were filtered through Whatman No. 1 filter paper. The acetone solvent was evaporated under reduced pressure using a a BUCHI R-210 rotary evaporator (Delaware, United States) and further freeze-dried (vertical freeze dryer: BK-FD 12S, Jinan, China) weighed, and stored at −20 °C.

### 2.4 Phytochemicals Screening

#### 2.4.1 Determination of total phenolic content

Total phenolic content (TPC) in the *Ulva spp* and *S.incisifolium* extracts were determined using Folin-Ciocalteu method [46]. Briefly, 20 μL of each sample (1 mg/mL) were mixed with 100 μl of 10 % Folin-Ciocalteu reagent followed by the addition of 80 μL of 8 % Na_2_CO_3_ and shaken gently. The solution was then incubated at room temperature for 30 min. The absorbance of the complex formed was recorded at 765 nm using FLUOstar Omega multimode microplate reader. Gallic acid (0-100 μg /mL) was used for the standard calibration curve. Results were expressed as gallic acid equivalents (GAE)/g dry weight of extract.

#### 2.4.2 DPPH free radical scavenging assay

The antioxidant activity of the sample extracts (*Ulva spp* and *S.incisifolium*) was studied using DPPH radical scavenging assay [47]. Briefly, equal volumes of each of the samples at various concentrations (0-50 μg/mL) were added to 40 µM DPPH solution (1:1) in 80 % methanol and the mixtures were shaken gently and allowed to stand for 30 min in the dark at room temperature. The blank and positive control (ascorbic acid) were prepared the same way without any extract. Ascorbic acid with varying concentrations of 0 to 50 μg/mL was used as standard and the absorbance was measured at 517 nm. The experiments were performed in triplicate and repeated three times. The antioxidant capacity was calculated using the following equation:

$$\%\;inhibition=\frac{Ab-As}{Ab}\times \;100$$

Where the Ab is the absorbance of DPPH without sample, As is the absorbance of the test sample containing DPPH solution.The 50 % inhibitory concentration (IC_50_) was calculated using linear regression analysis and compared to values obtained from ascorbic acid.

### 2.5 Characterization of the bioactive compounds of the seaweed samples by LC-qTOF-MS

The LC-qTOF-MS analysis was performed according to a previous method [48]. Briefly, 200 mg of each of the crude extract was dissolved in 3 mL water-methanol (50:50 v/v) and filtered with a filter membrane (25 mm diameter, 0.45 µm pore size). The separation analysis was performed using a Kinetex® C18 column of 6 × 150 mm, 100 Å, 5 µm particle size. AB Sciex® X500R QTOF coupled to an AB Sciex® Exion LC system was used for the high performance liquid chromatography (HPLC) quadrupole time of flight (HPLC-qTOF) high-resolution mass spectrometry analysis. The injection volume of the sample was 10 µL. The mobile phase consisted of two solvents: 1 mM ammonium formate in water as the aqueous mobile phase and 0.5 % formic acid in methanol as the organic solvent at a flow rate of 700 µL/min for a total run time of 35 min. The gradient elution started with a 2 % organic mobile phase and ended with a 98 % organic phase.

The operating conditions for the mass spectrometry (MS) analysis include electro-spray ionization (ESI) as the source type, positive ion mode, ion spray voltage (5.5 KV), the curtain gas made of nitrogen was set at 25 psi, the source temperature (450°C), declustering potential (80 V), collision energy (10 eV for MS and 20–50 eV for MS-MS scans) and ion source gases 1 and 2 (45 and 55 psi respectively.

#### 2.5.1 Identification of the bioactive compounds from the seaweed samples

The raw MS/MS spectra were first converted from the raw data (.mzML) format into Analysis Base File (.Abf) format using an ABF file converter (http://www.reifycs.com/ AbfConverter/index.html). Next, MSDIAL v4.9 software (http://prime.psc.riken.jp/compms/msdial/main.html) was used for compound spectra extraction, feature detection, peak alignment, ion species annotation, and spectral analysis, which generated a transition list with information on detected features including precursor *m*/*z,* MS^2^ ions and retention time [49]. The MSDIAL software identifies the metabolites in the crude seaweed extract by scanning them against the MS/MS natural compound database library available (https://mona.fiehnlab.ucdavis.edu/spectra/browse?query=). The MS-DIAL conditions were set as follows: mass range: 50–1500 Da, minimum peak height: 1000: amplitude, Mass slice width: 0.1 Da, MS^1^ accurate mass tolerance: 0.01 Da, MS^2^ accurate mass tolerance: 0.025 Da, MS/MS abundance cut off: 0, exclusion mass list: 0; Sigma window value: 0.5, identification score cut off: (80 %), Identification retention time tolerance: 100 min, spectral library: Fiehn/Vaniya natural product library – positive. (https://mona.fiehnlab.ucdavis.edu/spectra/browse?query=). The structural and molecular formula of the metabolites identified by the MSDIAL were further elucidated by the MSFINDER v3.2 software (http://prime.psc.riken.jp/compms/msfinder/download/repository/). The parameters settings are as follows: mass range: 50 -1500 Da, MS^1^ mass tolerance: 5, MS^2^ mass tolerance: 15, Abundance setting: 1 %, Element selection: O and N, Element ratio: 99.7 %, isotopic ratio: 20 %, Cut off spectral match: 80 %, Data source: COCONUT, PubChem, Natural Product Atlas, ChEBI, UNPD, NANPDP and Plant cyc. In addition, manual annotation of compounds was done using Metfrag (https://msbi.ipb-halle.de/MetFrag/), KNapSacK (http://www.knapsackfamily.com/KNApSAcK_Family/), and LOTUS (https://lotus.naturalproducts.net/) compound database [50].

### 2.6 *In silico* analysis

#### 2.6.1 ligand preparation

The two-dimensional (2D) structures of the 12 tentatively identified phytochemicals common to *S. incisifolium* and *Ulva spp* were retrieved from PubChem database (https://www.pubchem.ncbi.nlm.nih.gov) and saved in the sdf file format. LigPrep module of Schrödinger v2022 was used to prepare and process the 3D structure of the compounds. The operating parameters were as follows: energy minimization by Optimized Potentials for Liquid Simulations (OPLS-2005) force field, generate protonation state at a pH range of 7.4 ± 0, and considering all possible conformations of the ligand during the process [51]. Other parameters on the LigPrep module were kept as default. The generated library was subjected to a molecular docking- screening process.

#### 2.6.2 Receptor preparation

The raw crystal structure of *F. oxysporum* 14α-demethylase (PDB ID = 6CR2) was downloaded from the protein data bank (https://www.rcsb.org/structure/4BFX). The structure was prepared in Maestro v12.8 of Schrödinger v2022 using the Protein Preparation Wizard module (PPW) as described elsewhere [52]. Briefly, polar hydrogen atoms were added using PROPKA at pH 7.0 and water molecules were removed. The side loop region was refined, H-bonds were assigned, and OPLS-2005 force field was used to achieve the default restrained minimization at an RMSD value of 0.30 Å.

#### 2.6.3 Receptor Grid preparation and molecular docking

The receptor grid boxes for *F. oxysporum* 14α-demethylase were created using the receptor grid generation module, which involved establishing a bounding box around the centroid of the co-crystalized ligands associated with the receptor, specifically targeting the binding pocket. This approach guarantees that the ligands, once docked, will be constrained within the bounding box to prevent any non-specific binding [53]. The parameters for this process included a scaling factor of 1.0 and a partial charge cutoff of 0.25. The coordinates for the enclosing box were determined by selecting the centroid of the ligands along with their respective coordinates. Next, the prepared compounds were docked into the receptor's active site using the glide module, which involves a three-step docking process with increasing precision. The compounds were docked against the crystal structure of sterol 14-α-demethylase from *F. oxysporum* using extra-precision (XP) module.

#### 2.6.4 Molecular docking calculation

Molecular docking was executed to examine and understand the interaction between the receptor and the ligands. Hence, the Glide docking tool in Schrödinger Maestro which uses an OPLS_2005 force field for the calculations was used for this task. The interaction of the prepared ligands and *F. oxysporum* 14-α-demethylase were investigated by docking them into the active site of the receptor individually using the XP module. The 2D interaction diagram, poses, and their energies were further computed. Docking score threshold (≤ −8.0 kcal/mol) and MM/GBSA energy cutoff (≤ −40.0 kcal/mol) were selected based on established benchmarks for high-affinity.

#### 2.6.5 Prime MM-GBSA calculation

The free binding energy of the ligand-*F. oxysporum* 14α-demethylase complexes was calculated with specific parameters using the Molecular Mechanically Generalized Born Surface Area (MM-GBSA) as previously described [54]. Maestro's Prime module was utilized to ascertain the docked complex of Glide XP's MM-GBSA capacity.

### 2.7 Prediction of pharmacokinetic properties

QikProp is an advanced tool for predicting pharmacokinetic and physicochemical (ADME) properties of small organic molecules based on the full 3D molecular structure [55]. The drug-like properties of the tentatively identified metabolites were investigated using QikProp, a module of the Schrodinger software. Properties such as the molecular weight (acceptable range: 130.0–725 g/mol), Lipinski's rule of five (ROF), central nervous system (CNS, logBB and PSA), cell permeability (pCaco-2 and pMDCK), aggregation with serum albumin (logKhsa) and oral absorption (percentage Human Oral Absorption). The results obtained were compared with the average values obtained for 95 % of the drugs available in the software databases. The number of "stars" denotes the number of violations of these ranges of optimal values common to drugs, which are used as references/templates in QikProp. The properties, as well as their ranges of optimal values, were analyzed and presented below. Stars: 0–2 (high), 3 (medium), and > 4 (low); Percentage Human Oral Absorption ( %HOA): >80 % (high), 25–80 % (medium), and < 25 % (low); pCaco (intestinal cells): >500 nm/s (good) and < 25 nm/s (low); pMDCK (kidney cells): >500 nm/s (good) and < 25 nm/s (low); logKhsa (binding to human serum albumin): −1,5 (low) a 1,5 (high); CNS: −2 (low permeability) and > −2 (high permeability); logBB (blood/brain barrier): <−1 (low) and > −1 (easy permeation); PSA (Van der Waals surface area): >60 (does not cross the blood/brain barrier) and < 60 (to cross the blood/brain barrier); the number of permissible violations of Lipinski's rule of five (acceptable range: maximum is 4).

#### 2.7.1 Prediction of toxicological properties

Toxicological properties of the LC/MS identified seaweed compounds were determined using the admetSAR web-based tool at http://lmmd.ecust.edu.cn/admetsar1/predict/ since toxicity is the main task in developing novel therapeutic molecules [56,57]. Acute oral toxicity, Ames toxicity, carcinogenic properties, and rodent acute toxicity were predicted in the study.

### 2.8 Molecular Dynamics (MD) Simulation

MD simulations were performed to determine the stability of the protein-ligand complex. It was performed using the Desmond package of the Schrödinger Maestro suite with the OPLS-2005 force field. Three MD systems were created according to the method described by Fadaka and colleagues [58]. Firstly, the protein-ligand complex was solvated by enclosing it in a three-site transferable intermolecular potential (TIP3P) water box with the protein atom extending 10 Å beyond the box boundary. The entire system was neutralized by adding 0.15 M of Na^+^ and Cl^–^ counter ions. The systems were energy minimized and equilibrated at constant temperature and pressure (303.15 K and 1.01325 bar) using the Martyna-Tobias-Klein and Nose–Hoover thermostat as the default barostat method with an isotropic coupling style. The short-range method was used for the short-range interactions and analyzed with a cut off distance of 9.0 Å. The smooth particle mesh Ewald method with a cut-off distance of 12 Å was used for the long-range electrostatic interactions. The MD simulation was performed for 100 ns, and the internal energy was stored at an interval of 100 ps with 1000 frame numbers.

### 2.9 Statistical analysis

The experimental results of the TPC and DPPH assays are expressed as means ± SD of three replicates for each sample. One-way analysis of variance (ANOVA) was performed using graph pad prism 8.4, and significance levels were assessed at *p* < 0.05 using the t-test.

## 3 Results

**Fig. 1:**
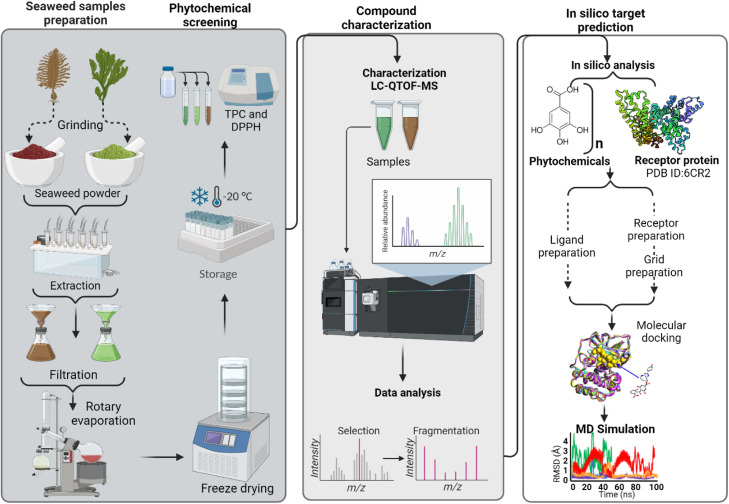
Flow chart summarizing the preliminary screening and LC-MS characterization of phytochemicals and insilico screening of potential antifungal compounds in *Ulva spp* and *S. incisifollium* extract.

The phytochemicals from the seaweed were extracted using acetone solution. Subsequently, the TPC and DPPH radical scavenging properties of *S. incisifolium* and *Ulva spp* extracts were determined. Untargeted LC-qTOF-MS/MS analysis was utilized to profile the phytochemical composition of the seaweed extracts, while molecular docking was conducted to evaluate the binding interactions between tentatively identified bioactive compounds and the target protein, *F. oxysporum* 14-α-demethylase. To provide a comprehensive overview of the experimental and computational workflow employed in this study, a schematic representation is presented in Fig. 1.

### 3.1 Sample yield and preliminary phytochemical screening

**Fig. 2:**
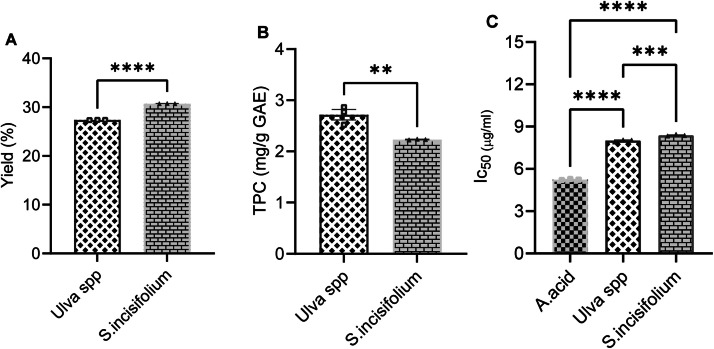
Percentage sample yield and preliminary phytochemical screening of the brown and green seaweed crude extracts. A. Percentage yield of the crude extracts. B. TPC content of the extracts. Student t-test. C. IC_50_ result of the DPPH analysis of the crude extract in relation to the standard ascorbic acid. **p= 0.0090, ***p=0.0005 and ****p<0.0001.

The powdered samples of the seaweed were exhaustively extracted with acetone solution. After the extraction, the acetone solvent was removed using a rotary evaporator and freeze-dried to obtain a dry powder sample. As illustrated in Fig. 2A, *S. incisifolium* exhibited a higher yield of 30.74 % compared to *Ulva spp*., with a yield of 27.38 %. Phenolic compounds are crucial plant constituents with redox properties associated with antioxidant activity [59]. Multiple hydroxyl groups in plant extracts are responsible for facilitating free radical scavenging. Based on this, the TPC of the seaweed plant extracts was evaluated using the Folin–Ciocalteu reagent. The results expressed in (GAE) per gram dry extract weight in Fig. 2B, show that *Ulva spp* had the higher TPC of 2.72±0.009 GAE/mg dry weight compared to *S. incisifolium* with 2.23±0.009 GAE/mg dry weight. In addition, the antioxidant activity of the extracts was evaluated using the DPPH radical scavenging assay, the inhibitory concentration are presented in Fig. 2C. The extracts had higher DPPH radical scavenging potency with an IC_50_ value of 8.01±0.07 µg/mL for *S. incisifolium* extract and 8.38±0.06 µg/mL for Ulva spp extract., compared to the IC_50_ value of 5.23±0.04 µg/mL for the positive control (ascorbic acid).

### 3.2 Profiling of phytochemicals in *S. incisifolium* and *Ulva spp* using untargeted QTOF LC-MS/MS Analysis

The crude acetone extracts of *Ulva spp.* and *S. incisifolium* were profiled by untargeted LC-qTOF-MS/MS in the positive ion mode to characterize potential secondary metabolites. The compound annotation was performed based on the outcome of LC-qTOF-MS/MS analysis and data from online databases. The base peak chromatograms (BP) of *Ulva spp* and *S. incisifollium* extract are presented in Fig.s 3 A and B, respectively. Each peak represents a potential compound or a group of compounds in cases where the compounds have the same or a close retention time. The result of the LC-MS enabled tentative identification of phytochemicals encompassing various classes, including terpenoids, alkaloids, naphthaquinone, small peptides carotenoids, phenolics, polyketides, and fatty acids. Terpenoids, alkaloids, fatty acids, and phenolics were the most abundant classes. Twenty-five and Twenty-nine bioactive metabolites were tentatively identified from and *Ulva spp* and *S. incisifolium*, respectively. A summary of the retention time, molecular formula, the mass-to-charge ratio (m/z), and the chemical classes of the crude acetone extracts are presented in the supplementary sections (Table S1 and S2), while Table 1 Table 1 highlights the key bioactive compounds identified in both seaweeds. In addition, it was observed that twelve compounds, namely corynanthine, gamma sitosterol, jasmonic acid, umbelliferone, cinnamic acid, sinapyl alcohol, ursolic acid, methylcinnamate, plumbagin, medicocarpin and myristic acid, are common to the seaweed species as shown in Fig. 4.

**Fig. 3:**
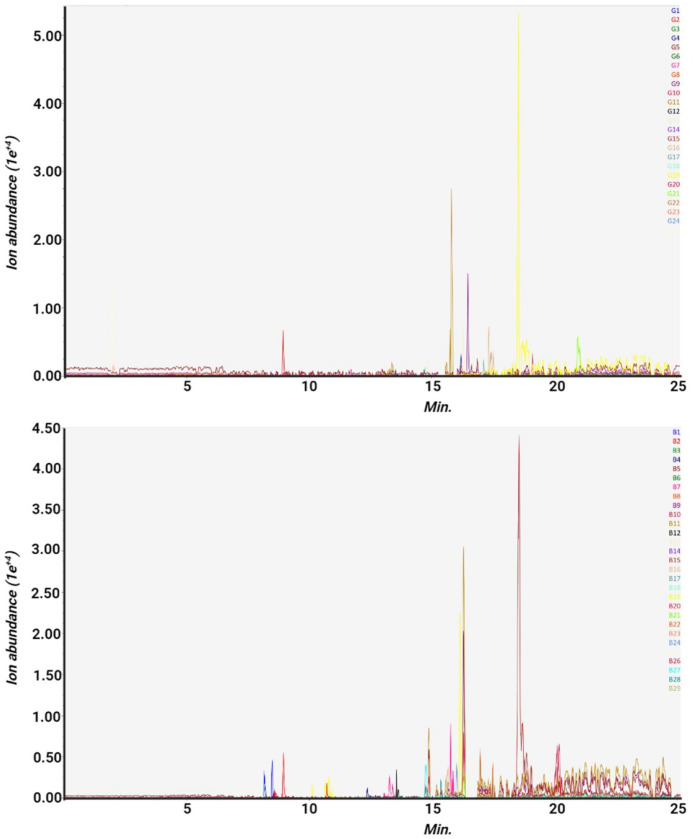
LC-MS/MS chromatogram of the acetone seaweed extracts. A. *Ulva spp* and B. *S. incisifolium*. Each color-coded chromatogram represents tentatively identified phytochemical.

**Table 1:** Summary of key bioactive compounds identified in both seaweed species and evaluated for antifungal potential.

No.	Compound	Chemical Class	Retention Time (min)	Molecular Formula	Observed m/z	Docking Score (kcal/mol)	MM-GBSA (kcal/mol)
1	Medicocarpin	Isoflavonoid	14.6 – 15.0	C22H24O9	433.1493	−13.63	−60.57
2	Corynanthine	Alkaloid	8.9 – 9.3	C16H29N5O5	372.2241	−10.60	−48.21
3	Merulinic acid A	Hydroxybenzoic acid	18.6 – 19.0	C24H38O4	391.284	−8.88	−44.36
4	Umbelliferone	Coumarin	15.5	C11H12O	161.0957	−6.42	−53.17
5	Plumbagin	Naphthoquinone	15.4	C11H8O3	189.0546	−6.04	−23.96
6	Sinapyl alcohol	Phenolic alcohol	13.5 – 14.0	C14H8O2	209.0598	−6.84	−11.18
7	Gamma-sitosterol	Terpenoid sterol	10.7 – 11.0	C24H44N4O4	453.3438	−10.53	−12.31

**Note:** Compounds 1–3 were selected for molecular dynamics (MD) simulations based on docking thresholds (Docking ≤ −8.0 kcal/mol; MM/GBSA ≤ −40.0 kcal/mol). Retention times may vary slightly between species. Full metabolite profiling is available in Supplementary Tables 1 and 2.

**Fig. 4:**
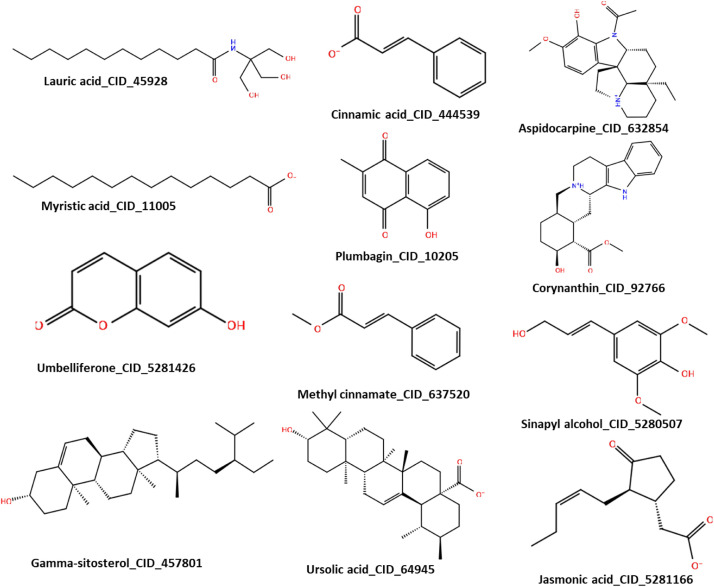
2D structural molecules of bioactive phytochemicals that are common in *Ulva spp.* and *S. incisifolium* as identified by LC-MS.

### 3.3 *In-silico* screening of tentatively identified compounds

#### 3.3.1 Molecular docking and selection of lead compounds based on binding affinity

Docking calculations were conducted on the twelve tentatively identified compounds common to *S. incisifolium* and *Ulva spp* to determine their binding interaction with the target protein, *F. oxysporum* 14-α -demethylase (6CR2). The 2D structures of the ligands are depicted in Fig. 4. The binding energies of all the common bioactive compounds, such as dock score and MM/GBSA are also represented in Table 2. The criteria for considering these bioactive constituents for further analysis were based on the docking score and MM/GBSA binding energies with the cut-offs ≤ −8.0 and ≤ −40.0, respectively. These were selected based on previous studies that identified similar values as indicative of strong and biologically relevant ligand-receptor interactions [[60], [61], [62]]. These thresholds help prioritize compounds with higher binding affinity and stability for further analysis."Gamma-sitosterol with a dock-score of −10.53 kcal/mol was excluded from the MDs because of the higher MMGBSA with a score of −12.31 kcal/mol. Aspidocarpin, sinapyl alcohol, umbelliferone, plumbagin, cinnamic acid, ursolic acid, myristic acid, jasmonic acid, and methyl cinnamate, did not meet the in-house set criteria of the energy cut-off and were excluded from the MDs analysis. From the docking analysis, medicocarpin, corynanthine, and merulinic acid met the set criteria with the dock-scores of −13.63 < −10.60 < −8.88 kcal/mol and MMGBSA energies of −60.57 < −48.21 < -44.36 kcal/mol, respectively and were subjected to MDs to understand their behavior in a biologically mimicked environment. Furthermore, the compounds performed better than the standard drug (propioconazole), which has a dock-score of −7.71 kcal/mol and MMGBSA energy of −10.71. A composite table with detailed interaction of the selected ligands, including the dock score, MM/GBSA, the number of hydrogen bond integrations, and types of residues and their distances (Å) are shown in Table 3. It is important to note that docking was performed using a single receptor conformation, which may not fully capture the dynamic nature of the protein. Future studies incorporating ensemble docking or induced-fit docking could provide a more comprehensive understanding of ligand binding.

**Table 2:** The binding energies of the bioactive compounds common to *S. incisifolium* and *Ulva spp*.

S/N	Compound	PubChem ID	Binding score	MMGBSA
1	Medicocarpin	44257429	-13.63	-60.57
2	Corynanthine	92766	-10.60	-48.21
3	Gamma-sitosterol	457801	-10.53	-12.31
4	Merulinic acid	5319371	-8.88	-44.36
5	Sinapyl alcohol	5280507	-6.84	-11.18
6	Umbelliferone	5281426	-6.42	-53.17
7	Plumbagin	10205	-6.04	-23.96
8	Cinnamic acid	444539	-4.91	-11.78
9	Ursolic acid	64945	-3.16	-59.39
10	Myristic acid	11005	-4.82	-39.15
11	Jasmonic acid	5281166	-3.78	-27.49
12	Methyl cinnamate	637520	-3.78	-1.26

All units are in kcal/mol.

**Table 3:** The binding properties of the three selected bioactive compounds after molecular docking calculations.

6CR2 complex	Dock score	Δ G Bind	H-Bond	H-bond residue interaction (Å)	Others
Standard	−7.71	−10.71	0	-	Pi Tyr122
Medicocarpin	−13.63	−60.57	4	Asn398, Thr96, 2x Phe234 (	−60.57
Corynanthine	−10.60	−48.21	1	Tyr136(1.98 Å)	SB (2) HEM
Merulinic acid	−8.88	−44.36	1	Tyr122	−44.36

Note: energies are in kcal/mol; SB: salt bridge.

**Fig. 5:**
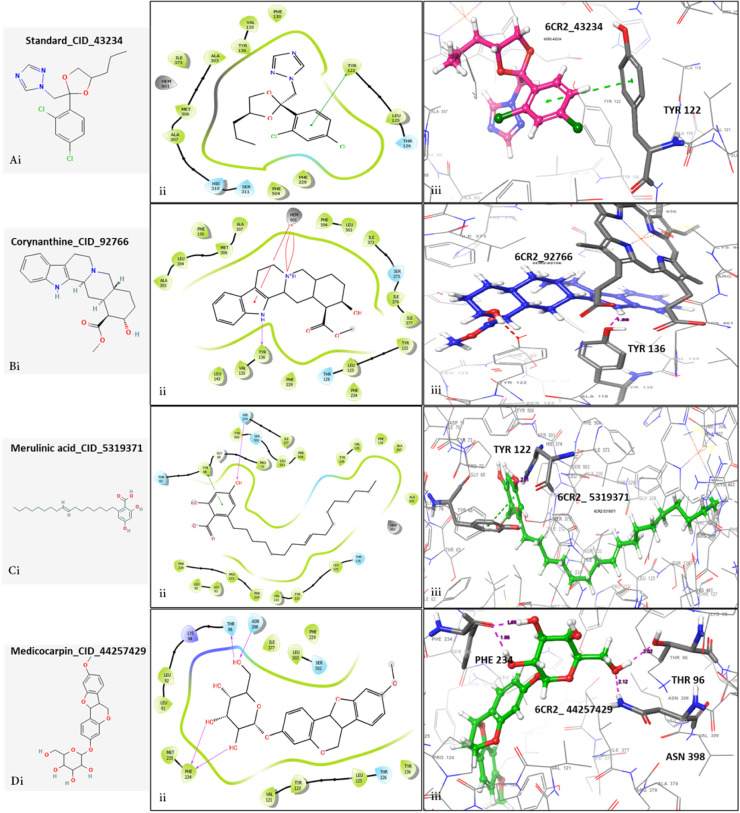
Molecular docking conformation of the complexes. A. Standard drug, B. Corynanthine, C. Merulinic acid, and D. Medicocarpin in the active site of *F. oxysporum* 14-α-demethylase protein (6CR2) showing pi-cation (green line) interaction, hydrogen bonding (solid pink arrow and broken pink lines) and hydrophobic interactions.

The molecular docking configuration for corynanthine as depicted in Fig. 5Bi, revealed that corynanthine showed a strong hydrophobic interaction with amino acid residues (Phe504, Met306, Val135, Tyr122, Ile377, Leu143, Ala307, and Phe234), one hydrogen bonding, and a pi-cation interaction with the heme prosthetic group of the target protein. The docking analysis of merulinic acid showed that this compound established three hydrogen bonds, a pi-pi cation interaction, and numerous hydrophobic bonds with residues of the target protein (Fig. 5Ci). The hydrogen bonds were observed between merulinic acid and active site residues Phe234, Asn398, and Thr96, while the pi-cation interaction is between Try68 of the target protein, *F. oxysporum* 14-α-demethylase. As observed in Fig. 5Di, the molecular docking configuration revealed that medicocarpin is buried in the hydrophobic internal cavity of the protein. The main driving forces involved in the binding of medicocarpin against amino acid residues of 6CR2 were predicted to be hydrophobic interactions (Phe229, Ile377, Tyr122, Leu503, Val121, Met235, Leu125, and Try136) and hydrogen bonding with Phe234, Asn398 and Thr96.

#### 3.3.2 ADMET properties of the identified bioactive compounds

Incorporating the drug-like predictions into development generates lead compounds with significantly higher chances of success in clinical trials. The ADME properties of the LC/MS-identified compounds are presented in Table 4. Furthermore, the toxicological characteristics of the compounds indicated that these molecules were Ames toxic, non-Ames toxic, non-cancer-causing, and showed feeble rodent acute toxicity properties (Table 5).

**Table 4:** Prediction of pharmacokinetic properties of molecules selected from the LC/MS analysis of brown and green seaweeds.

			Abs.	Distribution			CNS permeability			
ID	mol_MW	#stars	%HOA	PCaco-2	pMDCK	LogKhsa	CNS	LogBB	PSA	ROF
44257429	432.426	0	77.728	295.702	132.558	-0.49	-2	-1.463	125.013	0
5319371	390.562	4	81.633	40.367	19.591	1.135	-2	-2.807	89.741	1
92766	354.448	0	91.456	299.353	148.609	0.706	1	-0.083	70.746	0
637520	162.188	2	100	2317.411	1227.086	-0.17	0	-0.202	37.817	0
5281166	210.272	0	78.722	147.312	79.388	-0.346	-1	-0.844	75.469	0
457801	414.713	6	100	3383.137	1847.063	2.081	0	-0.358	22.453	1
444539	148.161	1	79.431	204.435	113.13	-0.514	-1	-0.556	51.632	0
5281426	162.145	0	81.115	623.262	296.765	-0.512	-1	-0.479	63.234	0
5280507	210.229	0	89.123	1050.261	521.639	-0.376	-1	-0.703	58.819	0
10205	188.182	0	79.548	478.168	222.851	-0.607	-1	-0.61	75.477	0
11005	228.374	3	95.771	235.332	131.719	0.294	-2	-1.295	51.079	0
64945	456.707	1	94.865	310.003	177.425	1.391	-1	-0.387	62.091	1

Note: ROF: Lipinski's Rule of Five; mol_MW: Molecular weight (g/mol); %HOA: %Human Oral Absorption; pCaco-2: intestinal cells; pMDCK: kidney cells; logKhsa: binding to human serum albumin; CNS: central nervous system; logBB: blood/brain barrier; PSA: polar surface area.

**Table 5:** Toxicity properties of the identified molecules from the LC/MS analysis of brown and green seaweeds.

ID	Ames toxicity	Carcinogens	Acute oral toxicity	Rat acute toxicity
44257429	AT	NC	III	2.2578
5319371	NAT	NC	II	2.6859
92766	NAT	NC	II	2.7324
637520	NAT	NC	III	1.781
5281166	NAT	NC	III	2.2974
457801	NAT	NC	I	2.6561
444539	NAT	NC	III	1.7416
5281426	NAT	NC	III	2.2141
5280507	NAT	NC	III	1.9179
10205	AT	NC	II	3.2704
11005	NAT	NC	IV	1.3275
64945	NAT	NC	III	2.3902

Note: AT: Ames toxic; NAT: Non-Ames toxic; NC: Non-carcinogenic; For acute oral toxicity: Category I: LD_50_ ≤ 50mg/kg, Category II: LD_50_ > 50mg/kg < 500mg/kg, Category: LD_50_ > 500mg/kg < 5000mg/kg, Category IV: LD_50_ > 5000mg/kg.

#### 3.3.3 MD simulations of Ligand–Protein complexes

**Fig. 6:**
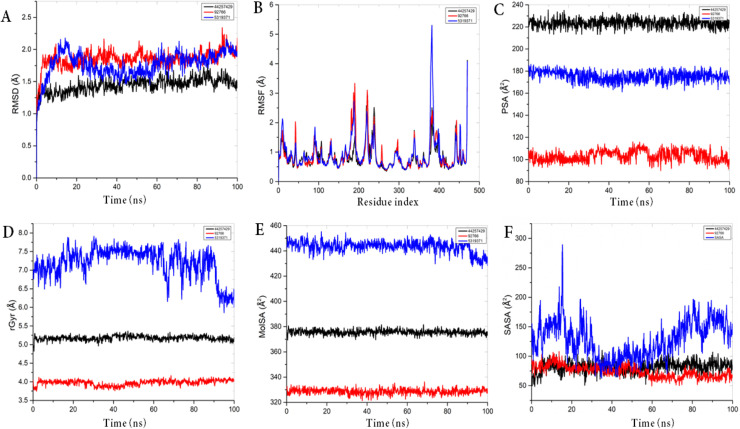
MDs of the top hit bioactive compounds common to the seaweed extracts and key target proteins. A. RMSD plot of the complexes in 100 ns, which is made up of α-carbon (Cα) atoms, throughout the simulations. B. RMSF plot of the hit complexes during the 100 ns MDs. (C–F) Compound-protein target interaction properties include C. PSA, D. rGyr, E. MolSA, and F. SASA. Each color represents different ligands with the receptor. Red: Corynanthine; Blue: Merulinic acid; Black: Medicocarpin, all complexed with *F. oxysporum* 14-α-demethylase.

MDs insights into structural and mechanistic information of complex interaction [58]. Based on the docking calculations, complexes were subjected to MDs to analyze the protein-ligand interaction and the stability mimicking a biological environment. The protein-ligand complexes were computationally simulated for 100 ns to decipher the dynamic behavior (Fig. 6 and Table 6). The root-mean-square deviation (RMSD) of the complexes were plotted against 1000 frame indexes for 100 ns (Fig. 6A). The root-mean-square fluctuation (RMSF) of the C-alpha of the protein complexed with these ligands was also plotted against the protein residues numbers (Fig. 6B). The ligand properties were also considered by plotting the radius of gyration (rGyr), molecular surface area (MolSA), solvent accessible surface area (SASA), and polar surface area (PSA) for the complexes against the frame index over the 100 ns simulation time (Fig. 6C-F). The simulation properties were calculated as the mean ± SD for RMSD, RMSF, and rGyr, MolSA, SASA, and PSA (Table 6). The RMSD profile of the medicocarpin-6CR2, merulinic acid-6CR2 and corynanthine- 6CR2 complexes showed an average deviation of 1.85 ± 0.13 Å, 1.76±0.19 Å and 1.44 ± 0.11 Å, respectively and was considerably stable until the end of the 100 ns simulations. This suggests that the complexes were stable throughout the simulation and experienced no substantial conformational changes. The fluctuations and internal motions were analyzed by calculating the RMSF of the protein binding site residues in the three complexes. The analysis as shown in Fig. 6B, revealed that slight fluctuations below 2.0Å were observed on the *F. oxysporum* 14-α-demethylase residues that interacted with the ligand's atoms of the three complexes within the first 100 ns of the simulations, however, higher fluctuations occurred afterward with merulinic acid-6CR2 having experiencing fluctuations above 5Å between amino acid residues 350 and 500. The rGyr Fig. 6C for corynanthine and medicocarpin was similar and relatively stable throughout the simulation, while slight fluctuations were observed with merulinic acid.

**Table 6:** Average values of the trajectory analysis of complexes over 100ns simulation time.

6CR2-Complex	RMSD	RMSF	rGyr	MolSA	SASA	PSA
Medicocarpin	1.44±0.11	0.82±0.42	5.17±0.06	375.65±1.88	81.46±9.7	223.44±3.82
Merulinic acid	1.76±0.19	0.91±0.58	7.2±0.40	443.37±4.64	128.21±30.9	175.45±4.60
Corynanthine	1.85±0.13	0.88±0.50	3.97±0.07	328.65±2.11	73.86±10.08	103.16±4.88

Values are Man ± SD.

The interaction of medicocarpin was mainly due to hydrogen bonds (Thr96, Phe234, and Asn398), hydrophobic contacts (Try122, Leu503, and Phe234), and water bridges (Leu92, Lys94, Val121, Ser 375 and Asn398). For corynanthine, the interaction was due to hydrogen bonds (Tyr122, and Tyr136), hydrophobic contacts (Val99, Try235, Phe239, Met242, Phe247, Phe254, Try257, Ile272, Ile276), and water bridges (Ser375). These interactions occurred for more than 30 % of the total simulation time. The interactions for merulinic acid showed hydrogen bonds, hydrophobic interactions, and pi-bonding. In summary, the MD simulation result showed that the three compounds were stable and interacted with the target protein during the simulation period. The results correlated well with the results obtained from the molecular docking analysis.

## 4 Discussion

The preference for natural compounds as a source of novel antifungal agents has increased drastically in the last decades. This is due to the increased resistance of fungi to the current pesticides, the negative impact of fungicides on non-target organisms and the pressing need to preserve the ecosystem [63]. Seaweeds, known for their diverse natural compounds, have been extensively utilized in preventing and treating various pathogenic infections [26,64]. Therefore, these seaweed-derived phytochemicals can be explored to discover novel antifungal agents. The current study determined the TPC and antioxidant potential *Ulva spp.* (green seaweed) and (*S. incisifolium* (brown seaweed). In addition, their phytochemical constituents were characterized using LC/MS. The *in-silico* section of this study aimed to elucidate the potential inhibitory and modulatory effects of bioactive compounds present in two species of seaweed against *F. oxysporum* 14-α-demethylase which is implicated in fungal metabolism.

Phenolic compounds, including flavonoids, tannins, lignin, and phenolic acids, are widely known for their strong antioxidant and broad antimicrobial activities, and marine algae have been reported to contain an abundance of these compounds [65]. Therefore, crude extracts from *Ulva spp.* and *S. incisifolium* were assessed for TPC. The findings in this study confirmed the presence of phenolic compounds in the crude seaweed extracts. This correlates with a previous study [66] that reported the presence of phenolic compounds in the crude acetone extract of select *Sargassum spp* obtained from the Queenscliff Harbour in Australia. Similarly, the research conducted by Ismail and co-workers [67] also confirmed the presence of phenolic compounds in *Ulva lactuca, S. muticum* and *S. acinarium* sourced from Hurghada Beach, Egypt. Although TPC values observed in this study exhibited slight variations compared to those reported in the literature, several factors such as harvest season, environmental conditions, geographical origin, or extraction methods could account for these discrepancies and influence phenolic content levels [68].

Given the reliability of the DPPH radical scavenging test, it is widely accepted for accessing the total antioxidant capacity of seaweed extracts [69,70]. Therefore, we evaluated the antioxidant capacities of acetone extracts from *Ulva spp* and *S. incisifollium*. In general, lower IC_50_ values signify higher antioxidant activity [71,72]. Both extracts had low IC_50_ values close to the standard ascorbic acid, which signified potent antioxidant activity. The antioxidant activity of these seaweed extracts might be attributed to the presence of phenolics and other bioactive compounds, including carotenoids, polyketides, fatty acids, and alkaloids, which possess the ability to scavenge free radicals, such as the DPPH radical, by donating hydrogen atoms or electrons to neutralize the unpaired electron in the radical [70]. These findings are consistent with a previous [71], that observed potent antioxidant activity in the crude extracts of four *Ulva* species (*Ulva intestinalis, Ulva clathrate, Ulva flexuosa* and *Ulva linza*). Similarly, acetone extract of *S. polycystum* and *S. duplicatum* [73] and *Ulva fasciata* [67] also exhibited excellent DPPH radical scavenging activity, correlating directly with their phenolic and flavonoid content. The DPPH analysis suggests that acetone extract of *Ulva spp* and *S. incisifollium* have potent radical scavenging activity.

Untargeted LC-QTOF-MS enables a comprehensive characterization of a wide range of compounds present in plant extracts. This facilitates the tentative identification of bioactive compounds with diverse structures and properties that may not have been previously identified or characterized [74]. The phytochemicals identified by LC-QTOF-MS in both algae can be categorized under diverse chemical groups; however, alkaloids, terpenoids, naphthaquinone, fatty acids, cinnamic acids, coumarins, and isoflavonoids groups were common to both *Ulva spp* and *S. incisifolium*. Alkaloids are naturally occurring nitrogenous-based compounds derived from amino acids [75]. Their potent antimicrobial activity is associated with their ability to accept or donate protons and form hydrogen bonds with proteins, enzymes, and receptors, resulting in growth inhibition [76]. They also possess ionizable groups, which enhance their bioactivity [77]. Terpenoids, coumarins, hydroxybenzoic acid, cinnamic acid, and isoflavonoids belong to a broader phytochemical group known as phenolic compounds [17]. Their antioxidative and antifungal effect can be attributed to the presence of an aromatic ring and a free hydroxyl group, which enhances their interactions with fungal cellular components via hydrogen bonding, metal chelation, membrane disruption, and oxidative stress induction [78]. Fatty acids are diverse molecules composed of long hydrocarbon chains with a carboxylic acid group at one end. Their anti-inflammatory and antifungal activities can be attributed to the presence of double bonds and the number and position of the hydroxyl groups [79]. Napthaquinones are naturally occurring phytochemicals with broad-spectrum bioactivities. Their antifungal can be attributed to their potent redox properties, allowing them to interact with essential cellular components, such as fungal DNA, lipids, and enzymes [80]. The LC-QTOF-MS result suggested that *Ulva spp* and *S. incisifollium* extracts possess diverse phytochemicals with potential bioactivities.

Molecules with therapeutic potential fail in clinical trials due to poor drug-like properties including absorption, distribution, metabolism, excretion, and toxicity. Accurate and timely detection using *in-silico* predictions of these properties can significantly reduce wastage of resources, further refines lead optimization efforts, and improving desired compound properties.

The molecular weight of all the bioactive compounds were < 500 g/mol which is within the standard range. Lipinski's RO5 predicts the probability of failure or success of potential therapeutic compounds. The lipinski RO5 for all the compounds shows that medicocarpin, corynanthine, methyl cinnamate, jasmonic acid, cinnamic acid, umbelliferone, sinapyl alcohol, plumbagin, and myristic acid did not violate any of the rules while merulinic acid, gamma-sitosterol, and ursolic acid showed one violation. All the compounds showed median to high values for oral absorption, median aggregation to plasma proteins, and high cellular permeability (pCaco-2 and pMDCK). While medicocarpin, merulinic acid, and myristic acid tend to not permeate the CNS, other molecules possess high CNS permeability. Considering the number of "stars", medicocarpin, corynanthine, methyl cinnamate, jasmonic acid, cinnamic acid, umbelliferone, sinapyl alcohol, plumbagin, and ursolic acid indicate high reliability, suggesting chemical similarity to known drug molecules of the QikProp software database compared to myristic acid with medium similarity and merulinic acid and gamma-sitosterol with low similarity. The toxicity result revealed that medicocarpin and plumbagin are Ames toxic among others which are non-Ames toxic. All together, they are not carcinogenic and showed no acute toxicity in rats. Overall, the generated pharmacological and toxicological results revealed that the bioactive compounds are all under the acceptable range, which signifies the druggability of the tentatively identified bioactive compounds.

The twelve bioactive compounds common to the species of the seaweed used in this study were subjected to molecular docking simulation and calculations against *F. oxysporum* 4-α -demethylase protein that is involved in fungal metabolism. A subset of three bioactive compounds exhibited improved docking scores with better intermolecular interactions with *F. oxysporum* 14-α-demethylase compared to the reference compound CID- 43234 (propiconazole). Notably, data from the docking calculation underscore the high binding affinities observed when the bioactive compounds of the seaweed interacted with *F. oxysporum* 14-α-demethylase protein. Specifically, medicocarpin had the highest binding affinity score followed by corynanthine and merulinic acid when compared to the reference compound. The observed differences in docking properties exhibited by these compounds may be attributed to their distinct interaction profiles with the amino acid residues located at the binding pocket of the target proteins .

Of note, amino acid residues contribute to the biological functionalities of target proteins. The visualization of interactions of the studied complexes revealed that these hit compounds were well fitted in the hydrophobic internal cavity of *F. oxysporum* 14-α-demethylase protein and the establishment of significant non-covalent bond interactions these assume pivotal roles in shaping the structure and stability of the ligand-receptor complex. These observations suggest the potency of these bioactive compounds against the selected target to inhibit fungal activity.

MDs investigates the dynamic behavior of biomolecular interactions in a biological system [81]. This is achieved through the measurement of the RMSD and RMSF. RMSD provides insights into the stability of the system while RMSF offers details on the flexibility and movement of specific residues over the period of simulation [82]. These parameters are important for understanding the function and behavior of molecular systems. The complexes of medicocarpin, corynanthine and merulinic acid with *F. oxysporum* 14- α -demethylase protein were individually subjected to MDs for a duration of 100 ns to ascertain their potential as promising therapeutic molecules. The RMSD profile is a plot of the RMSD in Amstrong unit (RMSD in A) against the period of simulation (time in ns) [83]. The RMSD of the three complexes displayed similar behavior throughout the simulation period with slight mean deviation. All complexes over a 100 ns MDs showed the stability and dynamic behavior of the compounds in the binding site of *F. oxysporum* 14- α - demethylase. In observation, the receptor remained relatively constant with negligible deviations for corynanthine, merulinic acid, medicocarpin, indicating that *F. oxysporum* 14-α demethylase structure maintains its integrity throughout the simulation time. The stability observed with the RMSD profile is correlated with a reliable simulation and because there were no significant protein conformational changes, it could be hypothesized that these molecules can be considered for therapeutic intervention against fungal activities.

The RMSF profiles of the complexes were plotted against the residue indexes of the receptor. The observed fluctuation peaks correspond to regions of the amino acids with higher flexibility. A high RMSF mean value indicates flexible loops or terminal that may be involved in binding interactions while RSMF profile with low values suggests rigidity with less fluctuation which is associated with the core structural elements of the protein [84]. Our data from the RMSF profiles of the three complexes suggested that specific protein residues exhibit considerable flexibility, which could be critical for the protein's functional conformational changes during binding interaction. These flexible regions may enhance good binding poses, allowing for induced-fit interactions that are essential for the biological function of the protein. Understanding these fluctuations is essential for elucidating the binding mechanism of medicocarpin, corynanthine and merulinic acid and their potential impact on the function of *F. oxysporum* 14- α- demethylase which can inform the design of targeted therapeutic strategies.

The RMSF profiles of the three complexes revealed similar patterns however, medicocarpin, exhibited the most favorable profile, with balanced flexibility that suggested efficient pose in the active site without compromising the structural integrity of the receptor. With lower RMSD, the RMSF data agrees by showing an optimal range of residue fluctuations essential for functional activity. This synergistic behavior suggested its potential as the most promising candidate for further investigation, underscoring the ability of medicocarpin to maintain a stable interaction with efficient protein dynamics for biological activity.

The radius of gyration (rGyr) is associated with the size and the compactness of the protein-ligand complexes [85]. The mean rGyr for the three complexes are in the order of merulinic acid > medicocarpin > corynanthine. The simulation pattern observed with the complexes showed that merulinic acid had the highest fluctuation profile compared with medicocarpin and corynanthine.

The molecular surface area (MolSA) in Å^2^ is plotted against the period of the simulation. This represents the surface area of ligand in the protein-ligand complex that is accessible to a probe with a radius 1.4 Å, which approximates the van der Waals surface area. MolSA focuses solely on the surface area of the molecule itself across simulation time. The three ligands maintained relatively steady MolSA till the end of the simulation, suggesting stability of their interactions with the protein.

The solvent-accessible surface area (SASA) is plotted against the period of the simulation. SASA takes into account the accessibility of the ligand's surface to solvent molecules. While corynanthine and medicocarpin ligands in the complexes maintained a steady SASA across the entire simulation time, suggesting good stability, there were some fluctuations seen in merulinic acid, which is indicative of slightly lesser stability.

The Polar Surface Area (PSA) in Å^2^, is plotted against the period of the simulation. PSA is calculated similarly to the solvent-accessible surface area (SASA) but focuses specifically on the portion of the surface area occupied by oxygen and nitrogen atoms and accessible to solvent molecules. Analyzing PSA often provides insights into the polar characteristics and surface properties of the ligands such as the hydrogen bonding interactions [86]. The mean PSA for the three ligands in the complexes is in the order of medicocarpin > merulinic acid > corynanthine. This corroborates the docking observations that medicocarpin formed a higher number of hydrogen bonding interactions compared to other ligands, contributing to its stable binding profile. The MDs data suggested that these molecules are promising candidates for further study as potential inhibitors of *F. oxysporum* 14- α -demethylase, with the potential for therapeutic application.

To our knowledge, this is the first time the antifungal effect of medicocarpin, corynanthine, and merulinic acid has been investigated. Nevertheless, it is noteworthy that several other natural compounds within their chemical groups (alkaloid, hydroxybenzoic, and flavonoid) have been reported to demonstrate antifungal activity by inhibiting ergosterol synthesis [[87], [88], [89]]. For instance, sanguinarine is an alkaloid that exhibited potent antifungal activity against *Candida albicans* by inhibiting ergosterol synthesis [90]. Magnoflorine, a natural alkaloid, was found to suppress the activity of 14- α -demethylase, inhibiting ergosterol synthesis and, consequently, hindering fungal growth [91]. Rutin, quercetin, and kaemferol are natural flavonoids that exhibited potent antifungal activity by repressing the activity of 14- α -demethylase and ergosterol synthesis [92]. Previous studies reported that hydroxybenzoic acid derivatives such as gallic acid and protocatechuic acid inhibited the growth of *Fusarium spp* [93,94].

*F. oxysporum*, like many other phytopathogenic fungi, can develop resistance to antifungal agents through several mechanisms [95]. These include mutations in the target enzyme (14-α-demethylase) that lower drug binding affinity, overexpression of efflux pumps that actively expel antifungal compounds, and upregulation of alternative metabolic pathways that bypass the inhibited enzyme [96]. Additionally, biofilm formation and epigenetic changes can further enhance the fungus's tolerance and adaptability [97]. However, unlike conventional single-target synthetic fungicides, the lead compounds identified in this study are structurally diverse and multifunctional. These natural compounds are known to possess multiple mechanism of action, making it more difficult for the pathogen to develop resistance .

For example, merulinic acid belongs to the phenolic acid class of compounds, which are known for their potent antifungal properties. Phenolic acids and flavonoids have been shown to exert antifungal activity primarily through chelation of essential metal ions such as Fe²⁺ and Cu²⁺, which serve as crucial cofactors for several enzymes involved in the ergosterol biosynthesis pathway [98,99]. By sequestering these metal ions, phenolic acids impair the catalytic activity of these enzymes, disrupting ergosterol production and compromising fungal cell membrane integrity. Additionally, phenolics may directly inhibit key enzymes in ergosterol synthesis pathway through specific interactions at the active site, further suppressing ergosterol synthesis and fungal growth [100]. Supporting this, Prasanna and colleagues reported that kaempferol, a flavonoid, binds to 14-α-demethylase via hydrogen bonding and hydrophobic interactions, thereby blocking ergosterol production and compromising fungal viability [101]. Similarly, cinnamaldehyde, a phenolic acid inhibited ergosterol synthesis by binding to the active site of 14 α-demethylase [102].

In addition, our in silico results from this study suggest that medicocarpin and corynanthine form stable interactions with 14-α-demethylase, potentially inhibiting ergosterol synthesis effectively. Given their novel structures and distinct binding modes compared to propiconazole (a known standard antifungal compound), these compounds may evade typical resistance mechanisms and provide a basis for next-generation antifungal agents.

## 5 Limitations and future directives

Limitations: While this study provides valuable insights into the antifungal potential of seaweed extracts, several limitations must be acknowledged. Firstly, the *in silico* nature of the assays does not account for the complexities of field applications where environmental factors can influence efficacy. The use of acetone for extraction, while effective, may not capture all potentially bioactive compounds present in the seaweeds. Additionally, although molecular docking and dynamics simulations offer predictive insights, they are based on theoretical models that may not fully represent *in vivo* interactions. The study's focus was also limited to a single fungal strain, which may not generalize across other pathogens. In addition, A key limitation of this study is the absence of in vitro antifungal assays for individual compounds such as medicocarpin. Future work should include MIC and zone of inhibition assays to validate the predicted antifungal activity and bridge the in silico-in vitro gap.

Future Directives: Future research should aim to validate the antifungal efficacy of these extracts in field trials to assess their practical application. Investigations into alternative extraction methods, such as supercritical fluid extraction or solvent-free techniques, could provide a more comprehensive profile of bioactive compounds. Additionally, expanding the study to include a broader range of fungal species and pathogens would help determine the generalizability of the findings. Further structural and functional studies are recommended to elucidate the precise mechanisms of action of identified compounds. 'Future work should include bioassays targeting ergosterol biosynthesis or enzyme inhibition to validate the predicted antifungal activity and bridge the in silico-in vitro gap. Finally, exploring synergistic effects between seaweed extracts and existing antifungal agents could enhance their efficacy and offer more robust solutions to combat fungal infections.

## 6 Conclusion

This study demonstrates the efficacy of *S. incisifolium* and *Ulva spp.* as a potential source of natural fungicides against *F. oxysporum*. LC-QTOF-MS analysis on the crude acetone extract of both seaweeds revealed the presence of diverse bioactive metabolites. *In-silico* studies uncovered substantial molecular interactions and remarkable stability of the ligand-protein complexes formed. Interestingly, a comparative evaluation between these bioactive compounds and an established standard drug (propiconazole) for this protein was conducted, yielding significant insights that underscore the importance of the study. This study not only sheds light on the therapeutic prospects of natural products from seaweed against fungal activity but also underscores the valuable contributions of our approach in elucidating complex interactions for drug discovery. Therefore, this study contributes to the potential use of the identified bioactive compounds (medicocarpin, corynanthine, and merulinic acid) as promising antifungal compounds. Further experimental validations are needed to confirm the antifungal efficacy of the identified compounds in practical agricultural settings.

## CRediT authorship contribution statement

**Omolola Aina:** Writing – original draft, Visualization, Software, Methodology, Formal analysis, Data curation. **Adewale O. Fadaka:** Writing – review & editing, Writing – original draft, Validation, Software, Resources, Methodology, Investigation, Formal analysis, Data curation. **Daniel Watson:** Writing – original draft, Visualization, Software, Methodology. **Cecilia Y. Ojemaye:** Writing – original draft, Software, Resources, Methodology, Investigation, Formal analysis, Writing – review & editing, Data curation. **Denzil R. Beukes:** Writing – review & editing, Writing – original draft, Methodology, Investigation, Data curation. **Kudakwashe Nyambo:** Writing – review & editing, Writing – original draft, Validation, Formal analysis, Data curation. **Kudzanai Tapfuma:** Writing – review & editing, Writing – original draft, Methodology, Data curation. **Vuyo Mavumengwana:** Writing – review & editing, Writing – original draft, Software, Data curation. **Nicole R. S Sibuyi:** Writing – review & editing, Writing – original draft, Validation, Supervision, Software, Project administration, Formal analysis. **Marshall Keyster:** Writing – review & editing, Writing – original draft, Methodology, Formal analysis, Data curation. **Ashwil Klein:** Writing – review & editing, Validation, Supervision, Writing – original draft, Software, Methodology, Conceptualization.

## Declaration of competing interest

The authors declare that they have no known competing financial interests or personal relationships that could have appeared to influence the work reported in this paper.

## Data Availability

The authors do not have permission to share data.

## Appendix Supplementary materials

Image, application 1
