# Supplementary material for: Sargassum incisifolium and Ulva spp metabolites activity and their molecular dynamics simulation against Fusarium oxysporum 14-alpha-demethylase
Source: Biotechnol Rep (Amst). 2025 Aug 20;48:e00919. doi: 10.1016/j.btre.2025.e00919 (PMC12446659; doi:10.1016/j.btre.2025.e00919)
Supplement: Supplementary file 1 [file mmc1.docx]

**Supplementary Material**

Table S1: Bioactive compounds identified from the crude acetone extract of *Ulva* *spp.* by LC-MS/MS in the positive ion mode.

| Chemical group | Rt | Compound | Formula | Observed m/z | Calculated m/z | Mass error |
| --- | --- | --- | --- | --- | --- | --- |
| Alkaloids | | | | | | |
| 1 | 8.65 | Mukonal | C_13_H_9_NO_2_ | 212.0697 | 212.071 | -4.2 |
| 2 | 8.93 | Corynanthine | C_16_H_29_N_5_O_5_ | 372.2228 | 372.224 | -3.5 |
| 3 | 9.3 | Argemonine | C15H25N5O5 | 356.1912 | 356.193 | -4.5 |
| 4 | 16.2 | Aspidocarpine | C16H30N6O4 | 371.2401 | 371.241 | -2.2 |
| 5 | 16.9 | Indole_3_aceticate | C22H14N2O4 | 371.1014 | 371.103 | -3.2 |
| 6 | 17.2 | Melatonin | C26H32N4O4 | 465.2502 | 465.25 | 1.28 |
| Fatty acids | | | | | | |
| 7 | 11.2 | Azelaic acid | C9H14O3 | 171.1014 | 171.102 | -0.6 |
| 8 | 12.4 | Jasmonic acid | C12H18O3 | 211.133 | 211.133 | 0.47 |
| 9 | 16.5 | lauric acid | C16H33NO4 | 304.2483 | 304.248 | 0.33 |
| 10 | 19.1 | Myristic acid | C14H28O2 | 229.2165 | 229.216 | 1.31 |
| Cinnamic acid | | | | | | |
| 11 | 13.3 | Methyl cinnamate | C10H10O2 | 163.075 | 163.075 | -1.8 |
| 12 | 13.5 | Sinapyl alcohol | C14H8O2 | 209.0598 | 209.06 | 0.48 |
| 13 | 14.9 | Cinnamic acid | C9H8O2 | 149.0593 | 149.06 | -2.7 |
| 14 | 16.9 | Ferulic acid 4-O-glucuronide | C_21_H_10_N_2_O_4_ | 355.0709 | 355.071 | -1.1 |
| Coumarin | | | | | | |
| 15 | 15.5 | Umbelliferone | C_11_H_12_O | 161.0957 | 161.096 | -2.5 |
| 16 | 17.3 | 6-Methylcoumarin | C_10_H_8_O_2_ | 161.0595 | 161.06 | -1.2 |
| Flavanoids | | | | | | |
| 17 | 17.1 | 3',4'-Dimethoxy-7-hydroxyflavanone | C12H14N4O_6_ | 311.0988 | 311.099 | 0.64 |
| Isoflavonoids | | | | | | |
| 18 | 14.6 | Medicocarpin | C22H24O_9_ | 433.1493 | 433.151 | 2.77 |
| Hydroxybenzoic acid | | | | | | |
| 19 | 18.6 | D8'-Merulinic acid A | C24H38O_4_ | 391.284 | 391.284 | -0.8 |
| Terpenoids | | | | | | |
| 20 | 10.7 | Gamma-sitosterol | C24H44N4O_4_ | 453.3438 | 453.344 | 0.66 |
| 21 | 21 | alpha-Tocopherol succinate | C33H54O_5_ | 531.4069 | 531.404 | 4.7 |
| 22 | 14.9 | Ursolic acid | C26H46O_6_ | 454.6408 | 455.337 | -3.7 |
| Small peptide | | | | | | |
| 23 | 1.88 | Betaine | C5H11NO_2_ | 118.0861 | 118.086 | -1.7 |
| Naphthoquinones | | | | | | |
| 24 | 15.4 | Plumbagin | C11H8O_3_ | 189.0543 | 189.055 | -1.6 |
| Polyketides | | | | | | |
| 25 | 17.4 | Phthalide | C8H6O_2_ | 135.0443 | 135.044 | 2.22 |

Note: Adduct = [M+H] ^+^ For Units: Retention time (Rt): minutes; Mass error: ppm.

**Table S2**: Bioactive compounds identified from the crude acetone extract of *S. incisifolium* by LC-MS/MS in the positive ion mode.

| Chemical group | Rt | Compound | Formula | Observed m/z | Calculated m/z | Mass error |
| --- | --- | --- | --- | --- | --- | --- |
| Alkaloids | | | | | | |
| 1 | 8.5 | Methylarmepavine | C_14_H_25_N_5_O_4_ | 328.1979 | 328.1964 | -4.57 |
| 2 | 8.9 | Corynanthine | C_16_H_29_N_5_O_5_ | 372.2241 | 372.2231 | -2.69 |
| Fatty acids | | | | | | |
| 3 | 15 | Montiporyne G | C_12_H_16_O | 177.1274 | 177.1272 | -1.13 |
| 4 | 12 | Jasmonic acid | C_12_H_18_O_3_ | 211.1328 | 211.1329 | 0.14 |
| 5 | 19 | Myristic acid | C_14_H_28_O_2_ | 229.2162 | 229.2166 | 1.71 |
| 6 | 20 | Palmitic acid | C_16_H_32_O_2_ | 257.2475 | 257.2476 | 0.36 |
| Cinnamic acids | | | | | | |
| 7 | 13 | Methyl cinnamate | C_10_H_10_O_2_ | 163.0754 | 163.0751 | -1.84 |
| 8 | 14 | Sinapyl alcohol | C_14_H_8_O_2_ | 209.0597 | 209.0595 | -0.96 |
| 9 | 15 | Benzaldehyde | C_7_H_6_O | 107.0491 | 107.0489 | -1.87 |
| 10 | 15 | Cinnamic acid | C_9_H_8_O_2_ | 149.0597 | 149.0593 | -2.68 |
| 11 | 15 | Coniferyl alcohol | C_10_H_12_O_3_ | 181.0859 | 181.0856 | -1.67 |
| Coumarins | | | | | | |
| 12 | 14 | Methylumbelliferone | C_10_H_8_O_3_ | 177.0546 | 177.0544 | -1.13 |
| Flavonoids | | | | | | |
| 13 | 18 | Quercetin-3-Arabinoside | C_27_H_50_N_2_O_2_ | 435.3945 | 435.3951 | 1.37 |
| Isoflavonoids | | | | | | |
| 14 | 15 | Medicocarpin | C_22_H_24_O_9_ | 433.1493 | 433.1505 | 2.77 |
| Hydroxybenzoic acid | | | | | | |
| 15 | 19 | D8'-Merulinic acid A | C_24_H_38_O_4_ | 391.284 | 391.2843 | -0.77 |
| Benzenoids | | | | | | |
| 16 | 16 | Piptamine | C23H41N | 332.3311 | 332.3307 | -1.2 |
| Terpenoid | | | | | | |
| 17 | 8.2 | Gibberellin A8 | C_19_H_25_O_7_ | 365.1595 | 365.1582 | -3.56 |
| 18 | 15 | Ursolic acid | C_26_H_46_O_6_ | 455.3367 | 455.3359 | -1.79 |
| 19 | 10 | Colupulone | C_19_H_34_N_4_O_5_ | 399.2602 | 399.2595 | -1.75 |
| 20 | 11 | Gamma sitosterol | C_24_H_44_N_4_O_4_ | 453.3435 | 453.3443 | 1.76 |
| 21 | 16 | Sargaol | C_27_H_38_O_2_ | 395.2945 | 395.2945 | 0.11 |
| 22 | 17 | Sargachromenol | C_27_H_36_O_4_ | 425.2686 | 425.2701 | 3.44 |
| 23 | 15 | Carveol | C_10_H_16_O | 153.1274 | 153.127 | -2.61 |
| 24 | 16 | α-Tochopherol | C_24_H_48_N_2_O_4_ | 429.3687 | 429.3688 | 0.23 |
| 25 | 16 | Tremetone | C_13_H_14_O_2_ | 203.1067 | 203.1064 | -1.48 |
| Carotenoids | | | | | | |
| 26 | 20 | Zeaxanthin | C_40_H_56_O_2_ | 569.4353 | 569.4334 | -3.35 |
| Apocarotenoids | | | | | | |
| 27 | 15 | beta-ionone | C_13_H_20_O_2_ | 209.1536 | 209.1533 | -1.43 |
| Naphthalenes | | | | | | |
| 28 | 15 | Plumbagin | C_11_H_8_O_3_ | 189.0546 | 189.0544 | -1.06 |
| Linear polyketides | | | | | | |
| 29 | 16 | Melophlin J | C_20_H_35_NO_3_ | 338.269 | 338.2682 | -2.36 |

Note: Adduct = [M+H] ^+^ For Units: Retention time (Rt): minutes; Mass error: ppm
